# In-game Social Interaction and Gaming Disorder: A Perspective From Online Social Capital

**DOI:** 10.3389/fpsyt.2020.468115

**Published:** 2021-02-03

**Authors:** Shupeng Heng, Huanfang Zhao, Minghui Wang

**Affiliations:** ^1^Department of Psychology, Faculty of Education, Henan Normal University, Xinxiang, China; ^2^Department of Psychology, School of Education, Henan University, Kaifeng, China

**Keywords:** in-game social interaction, online social capital, alienation, gaming disorder, moderated mediation model

## Abstract

**Background and Aims:** Social interaction in the online games has been found to predict gaming disorder, but little research has examined the mechanism of this association. Drawing on the social capital theory, the present study investigated the mediating role of online social capital on the relationship between in-game social interaction and gaming disorder and the moderating role of alienation on the relationship between online social capital and gaming disorder.

**Methods:** A sample of 457 Chinese massively multiplayer online role-playing game gamers was recruited to complete the In-game Social Interaction Questionnaire, Online Social Capital Scale, Alienation Scale, and Pathological Gaming Scale.

**Results:** The results showed that online social capital was a mediator in the relationship between in-game social interaction and gaming disorder. Moreover, for individuals with low alienation, the effect of online social capital on gaming disorder was weaker than for those with high alienation.

**Conclusions:** The present study provides new insight into the complex processes involved in the effect of in-game social interaction on gaming disorder, and the results have important theoretical and practical implications.

## Introduction

Playing online games has become a highly prevalent activity, which, in some cases, engenders negative consequences, and becomes addictive (1, 2). The 11th edition of the *International Classification of Diseases* [ICD-11; (3)] recently includes “gaming disorder, predominantly online” as an official diagnosis, and the fifth edition of the *Diagnostic and Statistical Manual of Mental Disorders* [DSM-5; (4)] also includes online gaming disorder as an emerging mental health issue that should be further investigated. At present, there is a lack of agreement as to the precise name and definition of the online gaming disorder, which always is referred to as game addiction (5), pathological online game use (6), or problematic online game use (7). The present study proposes to use the name gaming disorder, which means that excessive online gaming led to gamers developing addiction-like symptoms (e.g., overuse) and negative consequences on physical/psychological health (8–10). This term describes the quintessence of the phenomenon (i.e., the behavior is not only excessive but gaming-related problems) while avoiding the notion of dependency.

Past research showed that gaming disorder is associated with negative consequences on gamers' work, education, and their social relationships e.g. (11–14); the problem of gaming disorder and factors influencing it have received much research attention. Gamers' personality, motives, and psychosocial characteristics have been shown to be predictors of gaming disorder [e.g., (15–17)]. However, little research has addressed the influence of structural characteristics of online games, such as character play, leveling up, and in-game social interaction (18), which are critical design factors leading to engagement (19) and a greater risk for developing gaming disorder (18).

The current study focused on the in-game social interaction as a predictor of gaming disorder. Though previous studies have confirmed the direct link between in-game social interaction and gaming disorder (18, 20, 21), little is known about the mechanisms underlying the relation. Addressing this question is important for better understanding on how in-game social interaction influences gaming disorder (mediating mechanism) and when the link is most potent (moderating factors). The present study fills this gap by utilizing a large sample of Chinese massively multiplayer online role-playing game (MMORPG) gamers to test a moderated mediation model in which: (1) in-game social interaction increases online social capital, which, in turn, increases gamers' gaming disorder and (2) the indirect association between in-game social interaction and gaming disorder is moderated by individual factors such as alienation.

### In-game Social Interaction and Gaming Disorder

In MMORPG, social features mainly refer to collective play involving collaboration, community, and social interaction (22). Social interaction in MMORPG includes communication, cooperation, making friends with other gamers within the game context, belonging to a guild, clan, or group, and social support networks (23). Most MMORPGs encourage collective play and other forms of social interaction among gamers, which means that playing MMORPG is not a solitary activity but very much an intrinsically social activity (24).

The belongingness theory (25) suggests that people have a fundamental need to belong that motivates them to seek out social interactions and form close and meaningful relationships with others. The social features of video games provide opportunities for new meaningful and emotionally resonant relationships to develop, helping to satisfy the human need for affiliation and social support. Therefore, social need and developing online relationships are main motivations for online gaming (26), and the social elements of an online game shaped the gamers' desire to forge and maintain online relationships, which may play a considerable role in the initiation, development, and maintenance of gaming disorder (15). Consequently, the intensity of this social interaction has been known to be associated with gaming disorder (18, 20, 21).

### The Mediating Role of Online Social Capital

Online social interaction and relationships established in MMORPGs are based on collaboration and shared gaming experiences in which gamers exchange emotional or substantial support (22). Social capital is defined as the beneficial consequence (e.g., support-based resources) of social interactions and relationships (27), which can occur both offline and online (28) and is always separated into two subtypes: bonding and bridging (29).

Unlike other online friendships created merely by online communication (e.g., online social network services or SNS), these newly established strong ties in MMORPGs are more likely to generate social capital because of the frequent in-game social interactions and enjoyable social experience (22). Research has revealed that participation in guilds, quests, and inter-player interactions, engaging in clan/guild administration, joining in game-related groups, and the number of communication channels used for social interaction among gamers are positively associated with one's bridging and bonding social capital (22, 27, 30).

High online social capital is indicative of a meaningful and emotionally supportive online community (31, 32). However, social capital can also result in negative consequences. According to the uses and gratifications theory (33), individuals' dependency on media is related to use gratifications. Online social capital derived from in-game social interaction satisfies the need for affiliation and social support, which, in turn, leads to excessive gaming (34). Another mechanism through which online gaming may affect gaming disorder is suggested by the displacement hypothesis (35). Because of the “inelasticity of time” (36), playing online games takes away time from face-to-face interactions with one's offline ties (37), which can lead to the displacement of offline social contacts for online ties (38). Therefore, gamers who are absorbed with in-game social interaction may have an overall smaller and weaker offline social circle as a result of excessive online gaming (39). Reliance on online social interaction reduces offline contact (40), further maintaining online friendships and interactions. As gamers grow closer to their in-game contacts and their online social capital increases, offline activities become displaced and online game play becomes more desirable. Consequently, online gamers who participate in online social interaction might develop close ties with other gamers and receive social support from them, which, in turn, might lead to their psychological dependency on the online relationship (41), and the reduced levels of offline social interaction encourage the development of gaming disorder (42). Collins and Freeman (43) found that problematic video game play was associated with significantly higher online social capital and lower offline social capital. Therefore, the benefits of in-game social interaction, namely, online social capital, can increase the risk for problematic behavior in the form of gaming disorder.

Based on extant research, we believe that online social capital generated from in-game social interactions is an important predictor of problematic gaming, and thus we propose hypothesis 1: *Online social capital could mediate the relationship between in-game social interaction and gaming disorder*.

### The Moderating Role of Alienation

Although in-game social interaction may influence gaming disorder through the mediation effect of online social capital, it is possible that individuals are influenced differently by its effects. Therefore, it is necessary to examine moderators of in-game social interaction as it impacts gaming disorder. In the present study, we tested whether the direct and/or indirect association between in-game social interaction and gaming disorder was moderated by alienation.

Alienation is defined as the feeling of disconnectedness from social networks such as the family and peer group and an absence of social support (44). Individuals with high alienation often experience a sense of meaninglessness, helplessness, and loneliness (45). The compensatory Internet use model suggests that the Internet can provide opportunities for people to achieve some purposes that cannot be realized in real life (46). In the virtual online social environment, individuals can choose the groups to which they belong and gain opportunities to communicate with people based on their preferences, helping to compensate for the sense of helplessness and frustration experienced in real life (47). Therefore, university students with lower feeling of belonging to their surroundings may have been trying to compensate their need to belong *via* using excessive social networking. This result supported that belongingness is negatively associated with problematic social media use (48) and the relation between interpersonal dependency and gaming disorder (49). Thus, alienation appears to be an important risk factor for gaming disorder.

In addition, highly alienated individuals have difficulties in establishing effective connections with social groups and in maintaining relationships with others (45). According to the social skills deficit theory, individuals with a negative view of their own social competence are more likely to opt for the Internet to form and maintain their social relationships (50), and lonely individuals prefer online over offline interactions (51). Given that alienation may augment negative effect and increase the negative impact of behavior and environment on individuals (45), high levels of alienation might therefore serve as a risk factor that increases the potential negative effects of online social capital. In a sense, dependence on online social contacts for a sense of belonging may worsen offline social relationships, leading individuals to be more alienated from the offline social relationships maintained by traditional means of communication (52). Previous research has suggested that both offline and online social deficits are associated with gaming disorder, and offline social deficits can precede gaming disorder but then be exacerbated by the preoccupation with online social interactions and relationships (53).

Based on this review of the literature, we propose hypothesis 2: *Alienation would moderate the association between online social capital and gaming disorder. Specifically, the association would be stronger among individuals with high alienation than for those with low alienation*.

### The Present Study

**Figure 1:**
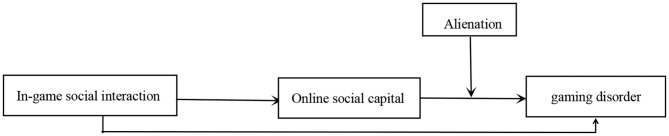
The proposed moderated mediation model. Mediation and moderation effects were controlled for gender and age.

Earlier research has established a link between in-game social interaction and gaming disorder. The current study expanded this research by testing the role of social capital and alienation in this relationship. First, this study examined the mediating role of online social capital in this relation; we expected that in-game social interaction would be indirectly related to gaming disorder through its effects on online social capital. Second, this study examined whether the indirect relationship between in-game social interaction and gaming disorder through online social capital would be moderated by alienation; we expected that the indirect association would be stronger for those with high alienation. These two research questions form a moderated mediation model, which can address both mediation (i.e., how does in-game social interaction lead to gaming disorder) and moderation (i.e., when is the effect most potent) as processes affecting the relationship between in-game social interaction and gaming disorder. According to previous studies that have demonstrated that gender and age [e.g., (54)] are associated with gaming disorder, gender, and age were included as control variables in the multivariate multiple regression model. Figure 1 illustrates the conceptual model.

## Materials and Methods

### Sample

Based on the comprehensive consideration of research accuracy and cost, a total of 495 MMORPG gamers were recruited through convenience sampling method from four universities in China during the 2018 fall semester. After excluding invalid questionnaires (with incomplete data), 457 valid questionnaires were collected (62.4% male, 37.6% female); the effective rate was 92.32%. Their ages ranged from 18 to 23 years old (*M* = 19.81, SD = 1.36). The participants completed a survey to collect information regarding demographic variables, in-game social interaction, online social capital, alienation, and gaming disorder.

### Measures

#### In-game Social Interaction

In-game social interaction often takes on the forms of communication with other gamers, guild, and group play (23). Therefore, it can be measured by the frequency of communication with other gamers and the frequency of group/guild actions. In addition, it is necessary to evaluate the respondents' attitudes toward the guilds/groups, the leaders, and other members of the guild/group (23).

Based on this, Zhong (22) developed an online game collective play scale which includes two factors (i.e., the frequency of collective actions and evaluation to the life in guild/group). This scale has yielded good construct validity and reliability (22). We modified this scale to measure in-game social interaction, which consists of two subscales: three items focus on the frequency of collective actions (How often do you communicate with other gamers while playing game? How often do you participate in group actions in a week? How often do you participate in guild actions in a week?) (α = 0.82) and were rated on a five-point scale (1 = never, 5 = always); the other three items relate to attitudes toward the life in guild/group (Are you satisfied with your guild/group? Are you satisfied with leader of the guild/group? Are you satisfied with members of the guild/group?) (α = 0.76) and were also rated on a five-point scale (1 = not satisfied at all, 5 = very much satisfied). Summing up the item scores created a scale score, with higher scores indicating higher levels of in-game social interaction. We checked the structural validity by a confirmatory factor analysis. All factor loadings were significant and bigger than 0.44, with indices indicating that the two-factor model fit well (RMSEA =.05, GFI = 0.94, AGFI = 0.91, CFI = 0.92), and two factors were significantly correlated with each other (*r* = 0.48, *p* < 0.01). Cronbach's α in the current study was 0.75.

### Online Social Capital

Online social capital was measured with a translated version of the Internet Social Capital Scales (55), which were modified to better fit the gaming context (e.g., Interacting with people in a game makes me feel like part of a larger community). Of the 20 items, 10 items focus on bonding social capital (α = 0.91), and the other 10 items relate to bridging social capital (α = 0.87). The participants responded to each question on a five-point scale (1 = strongly disagree, 5 = strongly agree). To establish structural validity, we conducted confirmatory factor analyses, and the indices indicated that the two-factor model fit well (RMSEA = 0.06, GFI = 0.96, AGFI = 0.93, CFI = 0.95). Cronbach's α for the online social capital scale was 0.96.

### Alienation

Alienation was measured by the 15-item Alienation Scale developed by Yang et al. (56). The scale consists of three subscales: sense of loneliness (e.g., I always feel lonely) (α = 0.83), alienation from family members (e.g., I have a sense of alienation from family members) (α = 0.79), and sense of social isolation (e.g., I feel like the people around me are like strangers) (α = 0.85). The participants rated each item on a five-point scale (1 = strongly disagree, 5 = strongly agree), with higher scores representing a higher sense of alienation. This scale has been validated in Chinese samples and has yielded good reliability (56). Cronbach's α for the alienation scale was 0.77.

### Pathological Online Game Use

A translated version of the 11-item Pathological Gaming Scale (6) was used to assess the level of gaming disorder. This scale was developed based on the DSM—IV criteria for pathological gambling (e.g., Do you become restless or irritable when attempting to cut down or stop playing video games?) The participants rated the symptoms on a three-point scale (1 = never, 3 = always). Summing up the item scores created a scale score, with higher scores indicating higher levels of game-related behavioral problems. The convergent and divergent validity, as well as the reliability of the original scale, are acceptable (6), and the translated version of the scale has demonstrated good reliability and validity in Chinese samples (57). Cronbach's alpha in the current study was 0.81.

### Procedure and Data Analysis

Surveys were conducted in classes by trained psychology graduate students after obtaining a written informed consent from participants. All participants were told that their participation was voluntary and that their privacy would be protected. The data collectors explained the requirements to the participants with standard instructions. At the end of the study, the participants were thanked for their participation. SPSS 21.0 was used for statistical analyses, descriptive statistics, correlational analyses, and examining the interaction effects and mediation effects.

### Ethics

The study was approved by the institutional review board of the Institute of Education, Henan Normal University, China. Written informed consent was obtained from all participants prior to assessment. This study did not involve human and/or animal experimentation and conformed to all guidelines according to the Declaration of Helsinki.

## Results

### Descriptive Analysis

The means, standard deviations, and Pearson correlational analyses for all variables are presented in Table 1. In-game social interaction was positively correlated with online social capital and gaming disorder (*r* = 0.39, *p* < 0.01; *r* = 0.18, *p* < 0.01). In addition, online social capital was positively correlated with gaming disorder (*r* = 0.43, *p* < 0.01). Finally, alienation was positively associated with gaming disorder (*r* = 0.47, *p* < 0.01).

**Table 1:** Means, standard deviations, and correlations for variables (*n* = 457).

**Variables**	***M***	**SD**	**1**	**2**	**3**	**4**	**5**	**6**
Gender	0.62	0.49	–					
Age	19.77	1.42	−0.02	–				
In-game social interaction	3.72	0.62	0.03	−0.01	–			
Online social capital	3.86	0.37	0.04	−0.04	0.39^**^	–		
Alienation	3.16	1.19	0.04	0.01	0.03	0.04	–	
Gaming disorder	1.92	0.56	0.02	−0.03	0.18^**^	0.43^**^	0.47^**^	–

*Gender was dummy coded such that 0 = female and 1 = male. False discovery rate method was used to correct p-values for multiple testing*.

^*^p < 0.05;

** *p < 0.01*.

### Testing for Mediation Effect

To test the mediation effect of online social capital in the relationship between in-game social interaction and gaming disorder, this study followed MacKinnon's (58) four-step procedure to test for significant associations between (a) in-game social interaction and gaming disorder, (b) in-game social interaction and online social capital, (c) online social capital and gaming disorder while controlling for in-game social interaction, and (d) a significant coefficient for the indirect path between in-game social interaction and gaming disorder *via* online social capital. We used the macro PROCESS (model 4) for SPSS (59) to examine the indirect effects. If the bias-corrected 95% confidence interval (CI) does not contain zero, the indirect effect is considered as statistically significant. This study included the participants' gender and age as covariates in all analyses.

Table 2 reports the results of the mediation analysis. Multiple regression analysis indicated that in-game social interaction was significantly associated with gaming disorder (β = 0.17, *p* < 0.001) and online social capital (β = 0.39, *p* < 0.001); when in-game social interaction was controlled, online social capital was significantly associated with gaming disorder (β = 0.42, *p* < 0.001). Finally, the bias-corrected percentile bootstrap method indicated that the indirect effect of in-game social interaction on gaming disorder through online social capital was significant, ab =.16, SE =.03, 95% CI= [0.11, 0.23]. Therefore, hypothesis 1 was supported.

**Table 2:** Testing the mediation effect of in-game social interaction on gaming disorder.

	**Model 1 (gaming disorder)**		**Model 2 (online social capital)**		**Model 3 (gaming disorder)**	
	**β**	***t***	**β**	***t***	**β**	***t***
Gender	0.03	0.34	0.05	0.53	0.02	0.14
Age	−0.02	−0.48	−0.03	−0.88	−0.01	−0.14
In-game social interaction	0.17	4.25^**^	0.39	8.04^**^	0.02	0.41
Online social capital					0.42	9.31^**^
*R*^2^	0.18		0.39		0.43	
*F*	6.23^**^		23.62^**^		25.76^**^	

*Gender was dummy coded such that 0 = female and 1 = male*.

^*^p < 0.05;

** *p < 0.01*.

### Testing for Moderated Mediation

We expected that alienation would moderate the mediation effect of online social capital on gaming disorder. According to Muller et al. (60), the parameters for the three regression models should be estimated to test the moderated mediation hypothesis. This study specifically estimated the moderating effect of alienation on (1) the relationship between in-game social interaction and gaming disorder (model 1), (2) the relationship between in-game social interaction and online social capital (model 2), and (3) the relationship between online social capital and gaming disorder as well as the residual effect of in-game social interaction on gaming disorder (model 3). The specifications of the three models can be seen in Table 3. In each model, we controlled for relevant covariates (gender and age). All the predictors were standardized to minimize multicollinearity (61). Moderated mediation is established if either or both of thesee two patterns exist (59): (a) the path from in-game social interaction to online social capital is moderated by alienation and/or (b) the path from online social capital to gaming disorder is moderated by alienation.

**Table 3:** Testing the moderated mediation effects of in-game social interaction on gaming disorder.

	**Model 1 (gaming disorder)**		**Model 2 (online social capital)**		**Model 3 (gaming disorder)**	
	**β**	***t***	**β**	***t***	**β**	***t***
Gender	−0.01	−0.12	0.02	0.43	−0.02	−0.35
Age	−0.03	−0.69	−0.04	−0.95	−0.01	−0.30
In-game social interaction	0.18	4.47^**^	0.39	9.33^**^	0.08	2.24
Alienation	0.48	11.92^**^	0.04	1.01	0.49	14.44^**^
In-game social interaction × alienation	0.07	1.87	0.06	1.45	0.01	0.23
Online social capital					0.34	8.87^**^
Online social capital × alienation					0.31	8.49^**^
*R*^2^	0.27		0.18		0.47	
*F*	34.59^**^		20.35^**^		59.20^**^	

*Gender was dummy coded such that 0 = female and 1 = male*.

^*^p < 0.05;

** *p < 0.01*.

As Table 3 illustrates, model 1 showed that both in-game social interaction and alienation significantly predicted gaming disorder (β = 0.18, β = 0.48, *p* < 0.01), but the interaction effect was not significant (β = 0.07, *p* > 0.05). Model 2 indicated that the main effect of in-game social interaction on online social capital was significant (β = 0.39, *p* < 0.01), but the main effect of alienation and the interaction effect were not significant (β = 0.04, β = 0.06, *p* > 0.05). In model 3, alienation and online social capital both had a significant effect on gaming disorder (β = 0.49, β = 0.34, *p* < 0.01), and the interaction effect of online social capital and alienation on gaming disorder was also significant (β = 0.31, *p* < 0.01).

**Figure 2:**
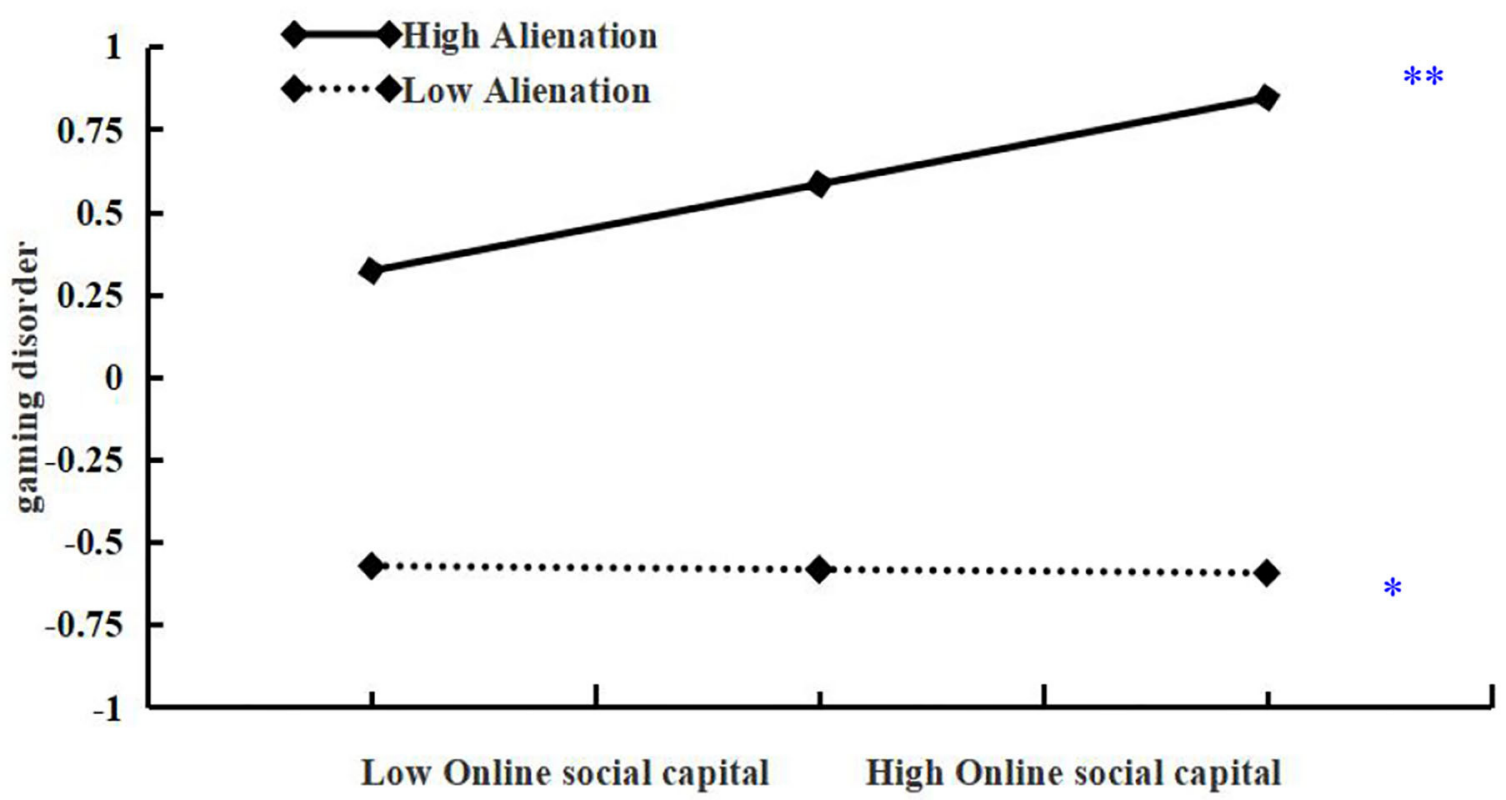
Alienation as a moderator of the relationship between online social capital and gaming disorder. The moderating effect is graphed for two levels of alienation: one standard deviation above the mean and one standard deviation below the mean.

To facilitate the interpretation of this interaction effect, we plotted predicted gaming disorder against online social capital separately for low and high levels of alienation (1 SD below the mean and 1 SD above the mean, respectively; see Figure 2). Simple slope tests showed that, for individuals with high alienation, higher levels of online social capital were associated with higher levels of gaming disorder (β_simple_ =.35, *p* < 0.001). However, for individuals with low alienation, the effect of online social capital on gaming disorder was still significant but much weaker (β_simple_ =.06, *p* > 0.05).

## Discussion

Although there is considerable empirical evidence of the impact of social features of online games on gaming disorder, the underlying mediating and moderating processes involved in this association have not been explored. The present study constructed a moderated mediation model to examine the mediating effect of online social capital in the association between social features of online game and gaming disorder and tested whether this indirect effect was moderated by alienation.

### In-game Social Interactions and Gaming Disorder

This study found that frequent in-game social interactions in MMOGs are positively related to gamers' gaming disorder. The results were largely consistent with previous research that underlines the importance of social features in gaming disorder (18, 20, 62). The findings could suggest that social interactions in video games have a significant influence on gaming disorder.

Social need is an important motivation for playing online games (26), and the commonly reported reasons for gamers' interest and motivation to play have been shown to be related to social interaction, such as “grouping together with others” and “being part of a guild” (21). The media system dependency theory (63) holds that the extent to which individuals depend on media is determined by the degree to which the media is useful in helping them to achieve their objective. Online games provide opportunities for new meaningful and emotionally resonant relationships to develop, helping to satisfy the human need for affiliation and social support. In addition, strong emotional bonds with fellow gamers may compensate for a lack of offline support and motivate further use (64). Consistent with this assumption, prior research showed that the social elements of an online game shaped the gamers' desire to forge and maintain online relationships but increased the risk for gaming disorder (62). The present study provides further evidence that searching the sense of fulfillment of human social need in MMORPG contributes to excessive game playing (34) that may further cause gaming disorder.

### The Mediating Role of Online Social Capital

Previous studies have shown that online social capital directly impacts SNS addiction (65) and problematic video game play (43). It could also mediate the relationship between SNS use and SNS addiction (66). In line with previous studies, the present study not only found that both in-game social interaction and online social capital positively predicted gaming disorder but also revealed the mediating effect of online social capital in the association between in-game social interaction and gaming disorder. Therefore, online social capital was the mechanism underlying the effect of in-game social interaction on gaming disorder, which supported hypothesis 1.

In addition to the overall mediation result, each of the separate links in our mediation model is noteworthy. For the first stage of the mediation process, our findings support the premise that the intensity of in-game social interaction is positively correlated with online social capital, which is consistent with the findings of extant studies (22, 64). This further supports the claim that online games appear to serve best as “third places” for informal sociability, where people are able to establish and maintain social ties by interacting and collaborating with strangers (28). MMORPGs encourage collective play and social interactions, which facilitate interdependent relationships, social interactions, and teamwork, all of which are beneficial for the gamers' social capital (22).

For the second stage of our mediation model, the present study found that online social capital was positively associated with gaming disorder, which suggests problematic gaming gamers' reliance on online social support networks. This finding is congruent with previous research claiming that developing online relationships is a main motivation for online gaming (26), and strong emotional bonds with fellow gamers may compensate for a lack of support offline and motivate further use (64). The results provide further evidence that online social capital may result in negative consequences. To some extent, by providing gamers an opportunity to build social relationships with other gamers and the group, MMORPGs are potentially addictive applications in the same way as social network services are (41).

### The Moderating Role of Alienation

Individual development is the result of an interaction between the effects of individual factors and environment (67). The present study tested the moderating effect of the individual factor of alienation in the relationship between online social capital and gaming disorder. The results showed that alienation was a risk factor of gaming disorder, which was in line with previous studies (51, 68–70). More importantly, alienation moderated the association between online social capital and gaming disorder.

The result showed that alienation from family, peers, and school was a significant and positive predictor of the level of gaming disorder. The primary socialization theory (71) suggests that individuals who have weak ties with prosocial institutions, such as family and school, often lack affiliation and turn to alternative social environments. For example, in one study, youth who were alienated from offline social relationships were eager to seek affection, friendship, and social support through participation in guilds and inter-player interactions (22). The virtual online environment provides more opportunity for communication and creates a sense of group identity, which provides a viable way for young people to seek a sense of belonging and to express their emotions (47). Individuals with higher alienation are often in a negative emotional state, such as estrangement, helplessness, loneliness, and meaninglessness (45). Studies have found that increased loneliness and lower social competence or greater social problems are associated with problematic game use (72, 73). These factors may increase the risk that alienated youth would excessively indulge in online gaming. Therefore, the effect of the Internet is a paradox because the social benefits of Internet use can have negative effects (74). The current results also showed that alienation enhances the facilitating effect of online social capital on gaming disorder. Compared with the online social capital of teenagers with a low level of alienation, the online social capital of highly alienated teenagers has a significant positive effect on gaming disorder.

According to the compensatory Internet use model (46), for individuals with higher alienation, excessive compensation for online social capital might displace offline social capital (35); the more they get from the online game, the more likely they are to rely on it and eventually show gaming disorder. This result is consistent with the “rich get richer” model: for those with more social support, using the Internet predicts better outcomes, but it predicts worse outcomes for those with less support (74).

### Limitations and Future Directions

The limitations of the present study should be addressed. First, we use only a single score as the score of the in-game social interaction in this study. In theory, in-game social interaction contains two dimensions (frequency and attitudes) (23), which has good content validity. However, “frequency” and “attitudes” cannot be considered as a single construct according to the results of data analysis. Therefore, the use of a single score may make the results of this study less stringent. In future research, “frequency “and “attitudes” should be used as independent constructs, and the scores of the two dimensions should be calculated independently instead of summing up two-factor item scores created as a single scale score, which will make the research results more rigorous. Second, the current study only examined the effect of online social capital on gaming disorder, but offline and online social capital are not mutually exclusive (75), and both of them may be related to gaming disorder. Future research should explore the fundamental social and psychological mechanisms that determine the relationship between combined online and offline social capital and gaming disorder. Third, in this study, we combine online bonding and bridging social capital as a single variable, but it is necessary to recognize that bridging and bonding social capital are not interchangeable (29). Therefore, future research may need to measure these two variables through a more valid and reliable method as well as explore how these different types of social capital are related to in-game social interaction, gaming disorder, and alienation. Fourth, future research is needed to explore other possible moderators and mediators that are important for refining our understanding of how in-game social interaction influences gaming disorder. Fifth, the sample of this study is comprised mainly of college students, with ages from 18 to 23 years; future research should focus on other age groups. Finally, our study was cross-sectional and cannot establish causality. Longitudinal data may provide a clearer understanding of the ways in which in-game social interaction can initiate, develop, and sustain online social capital and gaming disorder.

### Theoretical and Practical Implications

This research has several important theoretical implications. First, the present study provided further evidence that in-game social interaction can influence gamers' problematic video game play (15). Second, previous studies tended to regard social capital as a predictor of users' positive outcomes (31, 32), but the results of our study indicate that online social capital is a significant predictor of gaming disorder. Third, although previous studies have confirmed the relationship between in-game social interaction and gaming disorder (62, 64), there are few studies that capture the essence of why an in-game social interaction increases the risk of gaming disorder. The present study extended previous research by examining the joint effect of in-game social interaction, online social capital, and alienation on gaming disorder, providing a more comprehensive explanation of the mechanisms that explain how in-game social interaction influences gaming disorder.

Aside from the theoretical contributions, this research also has important practical implications. First, this study confirms that online gaming is a double-edged sword (76). For some adolescents, the benefits of in-game social interaction and capital may be offset by psychological dependency on online relationship and gaming disorder. Therefore, gamers should invest more time in offline social activities and maintain good social relationships with their family, friends, and other persons in the real world. Second, our findings can help practitioners understand the detrimental effect of in-game social interaction on gaming disorder through online social capital. This relationship is stronger for individuals with high alienation than for those with low alienation, which provides some implications for targeted interventions. The findings showed that online social capital was positively related to gaming disorder, which may be an indication that problematic game gamers rely heavily on online social support and may lack the social support needed in offline environments (77). Therefore, encouraging offline social capital in gaming disorder may be an effective form of prevention, and interventions should aim at improving offline social relationships and support. In addition, we should pay more attention to alienated teenagers, who are more susceptible to gaming disorder; interventions aimed at reducing their alienation may protect them from this risk.

## Conclusions

This study is an important step in unpacking how in-game social interactions relate to college students' gaming disorder. The findings suggest that the positive impact of in-game social interactions on gaming disorder can be partially explained by increased online social capital. Moreover, this indirect link was moderated by alienation in the second stage of the mediation process, such that the path from social capital to gaming disorder was stronger for more highly alienated individuals. This moderated mediation model is important because it provides a more comprehensive understanding of “how” and “when” in-game social interactions may increase gaming disorder.

## Data Availability Statement

The datasets generated for this study are available on request to the corresponding author.

## Ethics Statement

The study was approved by the institutional review board of the Institute of Education, Henan Normal University, China. Written informed consent was obtained from all participants prior to assessment. This study did not involve human and/or animal experimentation and conformed to all guidelines according to the Declaration of Helsinki.

## Author Contributions

All authors listed have made a substantial, direct and intellectual contribution to the work, and approved it for publication. MW make substantial contributions to the acquisition, analysis, or interpretation of data for the work.

## Conflict of Interest

The authors declare that the research was conducted in the absence of any commercial or financial relationships that could be construed as a potential conflict of interest.

## Supplementary Material

The data sheet and data analysis result presentation has been shared on Open Science Framework (https://osf.io/gnqf7/).
